# Decomposition of Gender Differences in Body Mass Index in Saudi Arabia using Unconditional Quantile Regression: Analysis of National-Level Survey Data

**DOI:** 10.3390/ijerph17072330

**Published:** 2020-03-30

**Authors:** Mohammed Khaled Al-Hanawi, Gowokani Chijere Chirwa, Tony Mwenda Kamninga

**Affiliations:** 1 Department of Health Services and Hospital Administration, Faculty of Economics and Administration, King Abdulaziz University, Jeddah 80200, Saudi Arabia; 2 Centre for Health economics, University of York, Heslington, York YO10 5DD, UK or; 3 Economics Department, Chancellor College, University of Malawi, Zomba, P.O. Box 280, Malawi; 4 Department of Social and Health Sciences, Millennium University, Blantyre P.O. Box 2797, Malawi; kamningatonymwenda@gmail.com

**Keywords:** body mass index, gender, differentials, Saudi Arabia, decomposition

## Abstract

Understanding gender differences in body mass index (BMI) between males and females has been much debated and received considerable attention. This study aims to decompose gender differentials in the BMI of people of the Kingdom of Saudi Arabia. The study decomposed the BMI gender gap into its associated factors across the entire BMI distribution by using counterfactual regression methods. The main method of analysis was newly developed unconditional quantile regression-based decomposition, which applied Blinder–Oaxaca decomposition using data from the Saudi Health Interview Survey. Gender differentials were found in the BMI, with females showing a higher BMI than males. The aggregate decomposition showed that both the covariate effect and the structural effect were significant at the 25th and 50th quantiles. Detailed decomposition indicated that income level and employment status as well as soda consumption and the consumption of red meat were significantly correlated in explaining gender differentials in BMI across various quantiles, but the magnitude varied by quantile. Our study suggests the government should consider introducing programs that specifically target women to help them reduce BMI. These programs could include organizing sporting events at the workplace and at the national level. Furthermore, the effect of soda consumption could be reduced by levying a tax on beverages, which might reduce the demand for soda due to the increased price.

## 1. Introduction

There is considerable disparity in body mass index (BMI) between men and women within and between countries. Several studies investigating these differential aspects in BMI have originated from developed countries [1,2,3]. There is mixed evidence pertaining to BMI distribution across females and males. While some studies appear to show that BMI is higher in males than in females [4,5,6], others show the opposite [7,8]. Gender-differential studies on BMI in low- and middle-income countries are scarce [9], despite a rapid increase in obesity, cardiovascular diseases [10,11], and other diseases associated with higher BMI. There is also a lack of information in the current literature on how differences in socioeconomic status in males and females explain differences in BMI distribution in the Middle East. This is despite the profound consequences associated with it, and existing evidence in other countries showing its role in exacerbating inequalities in health outcomes [12,13,14]. The role of socioeconomic status cannot be understated. Several studies have shown that differences in socioeconomic status have an increasing inequality effect on health outcomes such as diabetes [15,16]. 

Understanding the driving factors accounting for differences in BMI is important. Existing evidence suggests that the risk of non-communicable disease tends to increase with higher BMI [17,18,19,20]. Furthermore, an economic aspect is associated with BMI [21]. Costs from the treatment of obesity are higher, and as of 2014, the global economic impact of obesity was estimated to be USD 2.0 trillion, or 2.8% of the global gross domestic product [22,23]. Additionally, these effects may go beyond economic aspects and manifest into other consequences, such as loss of productivity due to loss of workdays and less productivity at work [24,25]. This implies that the difference in factors that drive the BMI gender gap is worth examining to ensure that factors that are likely to lead to the issues outlined above are thus addressed. 

Several studies have investigated the factors associated with BMI in the Kingdom of Saudi Arabia (KSA) [26,27,28,29,30]. These studies emphasize the relationship between BMI and other health outcomes. For example, an increase in BMI increases the risk of metabolic syndrome by more than three times, which is characterized by the relationships between metabolic, biochemical, and physiological factors [26]. In other aspects, an increase in BMI is the direct result of eating disorders that are propelled by varying attitudes, leading to problematic feeding behavior [27]. Diet also plays an important role in affecting BMI [31]. Aspects such as excessive drinking of soda and eating red meat were found to be associated with higher BMI [32,33,34,35]. In another study, Al-Qahtani [30] demonstrated that men have higher BMI than women, and that, across various income quintiles, the risks of being overweight or obese increase with an increase from low- to high-income quintiles. Although the situation is identical, the above-mentioned studies differ from our study in terms of both methods and focus.

The KSA provides a conducive case study to understand the current phenomena. First, in recent times, there have been changes in the laws that govern lifestyles and the workplace for both males and females; this may or may not have some implications on the BMI of the two groups. For example, Al-Hazzaa and Musaiger [36] found that healthy lifestyle has deteriorated due to the availability of automobiles, and high-fat and high-calorie food. This lifestyle change has also led to a change in the pattern of diseases, from communicable to non-communicable diseases, including hypertension and diabetes [37]. Second, the rapid Westernization of the healthcare system in the KSA could also, to some extent, be a factor that contributes to the difference in the BMI, given that men and women have different healthcare-seeking habits [38]. Third, as statistics show, gender differences in BMI in the KSA are quite vast, with females being significantly more obese (44%) than males (26.5%) [39]. Indeed, non-communicable diseases account for 73% of all deaths, from which the majority are cardiovascular diseases (37%), cancer (10%), and diabetes (3%), to mention a few, which are associated with differences in BMI across the population [40].

Therefore, the current paper contributes to the existing literature on gender gaps in the BMI by investigating how socioeconomic status may affect BMI inequality across gender. Our paper departs from previous research in various ways. We decomposed the BMI gender gap into its associated factors across the entire BMI distribution by using counterfactual regression methods [41,42]. This is the first paper to adopt such a technique in the KSA and neighboring countries, where the BMI issue is much more prevalent. Distributional decomposition is essential because using mean differences, which is at one point of the distribution, may not be representative of differences in other portions of the distribution that might be of interest [9,43]. This method has widely been employed in previous research, such as on health inequalities [44] understanding cross-country differences in BMI [1], and returns to wages [45,46]. Additionally, the study used national representative data from the KSA that are unique to the country. Specifically, we answered the following questions: (1) Is the BMI for males and females different in the KSA across the entire BMI distribution?; (2) How do socioeconomic factors such as age, education, and employment status contribute to the differences in BMI distribution?; (3) How much of the difference in BMI distribution is accounted for by diet factors such soda intake and red meat consumption?

## 2. Materials and Methods

### 2.1. Data Source and Sample

This cross-sectional study used data from the Saudi Health Interview Survey (SHIS), which is a national representative survey conducted in 2013 and carried out collaboratively by the Saudi Ministry of Health (MOH), the Institute for Health Metrics and Evaluation (IHME), and the University of Washington, Seattle, Washington, USA. The SHIS is a rich source of information, as it collects data on health and demographic characteristics with the aim of establishing the extent of chronic conditions and their risk factors. A multistage stratified probability sampling method was used to recruit survey respondents to ensure that the findings of the survey were representative of the Saudi population. The Census Bureau of the KSA divided the Kingdom into small clusters of households (averaging about 140 households in each cluster) and labeled them as enumeration units. These enumerations units serve as primary sampling units (PSUs) for the survey. The number of households within each PSU depends upon the population size, density, and geographical distribution. A probability proportional to the size sample of PSUs was randomly selected from each of the 13 administrative regions. Fourteen households from each PSU (enumeration unit) were randomly selected and contacted [47]. In total, 12,000 households were originally contacted, and 10,735 individuals aged over 15 years were interviewed, with a survey response rate of about 90%. A detailed description of the sampling methodology and data collection is available elsewhere [48,49]. We restricted our analysis to only respondents with no missing observations for any of our variables of interest, thereby reducing our analytic sample to a total of 7746 respondents (the analytic sample). Of the sample, 4128 (53.29%) were men and 3618 (46.71%) were women.

### 2.2. Variables

Our dependent variable was the BMI, which was computed from an anthropometry module that included data on weight and height, among others. In terms of definition, BMI is the ratio of an individual’s weight in kilograms divided by the square of the height in meters (kg/m^2^) [50]. In the survey, BMI was objectively measured by trained medical practitioners. It was collected as part of the anthropometry module for the survey biomarkers. In the analysis, we used the natural log of BMI. We used a natural log to scale down the weight of the variables. Additionally, a natural log allowed us to normalize our BMI variable and reduce the effects of outliers.

The independent variables were based on previous studies that indicated factors associated with the BMI [1,51,52,53,54,55,56]. To measure socioeconomic status, we used the grouped monthly income in six categories: less than Saudi riyal (SR) 3000 (reference category), SR 3000 to less than 5000, SR 5000 to less than 7000, SR 7000 to less than 10,000, SR 10,000 to less than 15,000, and SR 15,000 or more.

Given that education is also an important factor, we controlled for education using the following groups: below primary school (reference category), primary school, intermediate school, high school, and higher education (college/university +). Similarly, the age variable was split into categories: 15–24 (reference category), 25–34, 35–44, 45–54, and 55 years or above. Gender was coded as 1 if male and 0 if female. Marital status was also captured as a binary variable, where a value of 1 was assigned if married and 0 was assigned otherwise. We also controlled for the thirteen administrative regions, namely, Almadina Almonawra, Albaha, Aljouf/Quriat, Aseer/Bisha, Eastern Region, Haiel, Jazan, Najran, Northern Borders, Qaseem, Riyadh, Tabouk, and the Western Region.

### 2.3. Data Analysis

This study used unconditional quantile regression (UQR) [41,42]. The goal of UQR is to decompose an observed difference at the τth quantile point into two parts: the part attributable to the difference in the association between BMI and covariates (structural effect at the τth quantile), and the part reflecting the difference of covariate distributions (covariate effect at the τth quantile).

Suppose the quantile distribution of male and female BMI is given as $q\;\left({\mathrm{BMI}}_{f}\right)$ and $q\;\left({\mathrm{BMI}}_{m}\right).$ The difference in distribution may be expressed as

(1) $$q\;\left({\mathrm{BMI}}_{f}\right)\;-\;q\;\left({\mathrm{BMI}}_{m}\right)$$

Let counterfactual distribution for the females be $q\;\left({\mathrm{BMI}}_{c}\right)$ (i.e., BMI distribution if they had male characteristics). Thus, the difference in the BMI between females and males may be presented in terms of the counterfactual as

(2) $$q\;\left({\mathrm{BMI}}_{f}\right)\;-\;q\;\left({\mathrm{BMI}}_{m}\right)=\left[q\;\left({\mathrm{BMI}}_{f}\right)-q\left({\mathrm{BMI}}_{c}\right)\right]+\left[q\;({\mathrm{BMI}}_{c}-q\left({\mathrm{BMI}}_{m}\right)\right]$$

where $q\;\left({\mathrm{BMI}}_{f}\right)-q\left({\mathrm{BMI}}_{c}\right)$ is the covariate effect, and $\left[q\;({\mathrm{BMI}}_{c}-q\left({\mathrm{BMI}}_{m}\right)\right]$ is the coefficient effect. Using recentered influence functions (RIFs), Equation (2) can be expressed as

(3) E (RIF (BMI|qτ)= Xβ

where β is the unconditional effect of covariate X on quantile τ of BMI distribution. Applying Blinder–Oaxaca decomposition to the above equation yields

(4) $$\hat{q_{\tau \;}}\left({\mathrm{BMI}}_{f}\right)-\hat{q_{\tau \;}}\left({\mathrm{BMI}}_{m}\right)=\left[\bar{X_{f}}(\hat{\beta_{c}}-\hat{\beta_{f}})+\;e\right]+\left[\left(\bar{X_{m.\;}}\hat{\beta_{m\;}}-\bar{X_{f.\;}}\hat{\beta_{c\;}}\right)+\varepsilon \;\right]$$

where $\hat{q_{\tau \;}}\left({\mathrm{BMI}}_{f}\right)-\hat{q_{\tau \;}}\left({\mathrm{BMI}}_{m}\right)$ is the BMI differential at the τth quantile, and X represents the covariate averages, $\bar{X_{f}}(\hat{\beta_{c}}-\hat{\beta_{f}})$ is the coefficient effect, and $\left(\bar{X_{m.\;}}\hat{\beta_{m\;}}-\bar{X_{f.\;}}\hat{\beta_{c\;}}\right)$ is the covariate effect. Variables e and ε are errors resulting from the estimation of the coefficient and covariate effects, respectively. A significant structural (coefficient) effect means that there are some other factors beyond for what we controlled, which explains the BMI differential in males and females in the KSA.

### 2.4. Ethical Clearance

In this study, we used records on aspects such as weight, education, age, height, income, diet behavior, and regions of residence. Data collection, archiving, and use were performed in compliance with the World Medical Association Declaration of Helsinki. The study protocol was approved by the Saudi Ministry of Health and its Institutional Review Board (IRB). The participants consented and agreed to participate in the study. Two instances of verbal consent were taken at the household and individual level. Only those respondents who gave their consent were recruited for the study. Participants were also informed that taking part in the study was voluntary, and that they could withdraw at any time without giving any reason. At the consent stage, all participants were also informed that the collected data would be used in future research. All personal identifiers were removed from the data to allow for secondary data use. The MOH and the IHME granted permission to use the data; thus, no further clearance was necessary, as this was done at the data-collection phase.

### 2.5. Data Availability Statement

The datasets generated and/or analyzed during the current study are not publicly available due to privacy, confidentiality, and other restrictions. Access to data can be gained through the MOH in Saudi Arabia.

## 3. Results

### 3.1. Social and Demographic Characteristics

Table 1 presents summary statistics of the social and demographic characteristics in the sample stratified by gender. We tested for the differences in the characteristics of males and females. Since BMI is a quantitative variable, we used group t-test for unequal variance. This test is used to compare if the mean of a continuous variable is the same between two groups. For categorical variables, we used the Chi-squared test. For consistency, we only reported the *p*-values for all the tests. Data showed that the BMI between males and females was statistically different (1.152, *p* < 0.001), with females having a slightly higher BMI (29.12) than males (27.97).

Furthermore, according to Table 1, in terms of marital status, 70% of males in the sample were married, while only 64% of females were married. The difference in the proportion of married men and women was statistically significant (*p* < 0.001). Furthermore, more males had primary and secondary levels of education than females. However, the proportion of postsecondary education attainment was equal, at 26% for both males and females. In addition, more females (44%) in the sample had no significant formal education, which was comparatively higher than for males (11%), and the difference was statistically significant (*p* < 0.001). Regarding employment, 67% of females were employed as compared to 63% of males, and the difference was statistically significant (*p* < 0.001).

According to Table 1, age representation in the sample showed an inverted U shape for both males and females as depicted by the initial increase followed by the decrease. As observed, this was predominantly increased at the age group of 25 to 34 years for both males and females, with percentages decreasing as age increased. Further, data showed that the average number of days of male soda consumption was higher (4.24) than that of females (3.68) (*p* < 0.001). Similarly, average male red meat consumption was about 3.42 days, compared to 2.90 days for females (*p* < 0.001). In terms of income groups, data showed that, for both males and females, there was a higher concentration in the lower-income groups than in the range of SR 5000 to 10,000. This concentration decreased as income increased, with the last group (SR 15,000 or more) having the lowest percentages of people (13% for males and 11% for females) and the differences between males and females at this group were statistically significant (*p* < 0.001).

Moreover, in terms of regions, as shown in Table 1, approximately 16% of males and females were from the Western region, which represents the highest number of participants. The second largest contributor in terms of participants in the sample was Riyadh, with approximately 14% of male and female participants coming from this region. Qaseem and Aljouf/Quriat were the lowest contributors (only 4%) of female participants to the sample, while Qaseem contributed only 3% of the male participants, the lowest among all regions.

**Figure 1:**
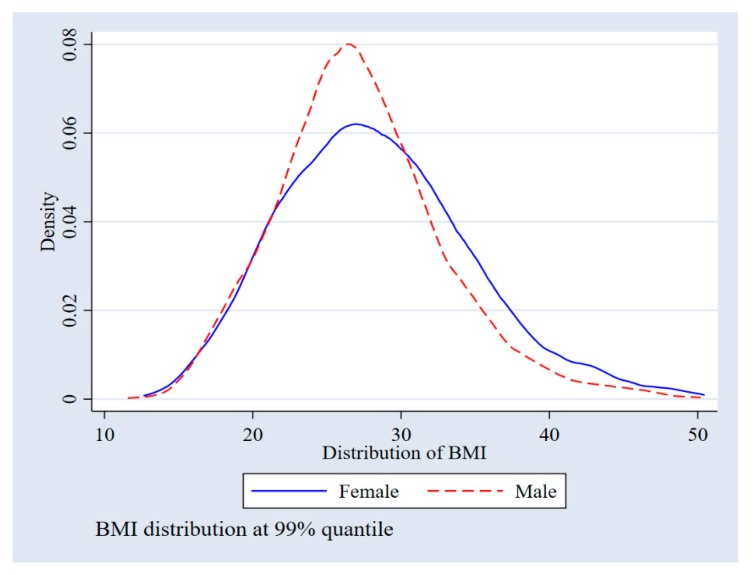
BMI gender distribution. Results from the 2013 Saudi Health Interview Survey (SHIS).

We further examined the BMI–gender relationship across BMI distribution in Figure 1. There was no clear pattern in terms of the difference in BMI distribution in the lower tail; the difference became visible beyond a BMI of 22 (this value is not to be confused with a statistical cut-off, rather, it shows where the difference for the current sample begins). Thereafter, female kernel density dominated male distribution, suggesting that females have a higher BMI than males, above the cut-off BMI of 22. Thus, this confirmed that the use of mean-based regressions, such as ordinary least squares (OLS), may indeed miss important information.

**Figure 2:**
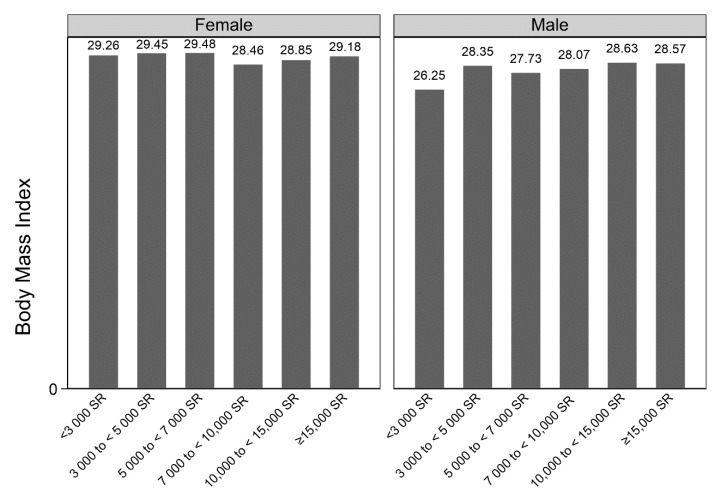
BMI distribution across income by gender. Results from the 2013 Saudi Health Interview Survey (SHIS).

Figure 2 presents a graphical presentation of the BMI across income quintiles between males and females in the sample. There was a general increase in BMI as income levels increased for males, starting from the income quintile of SR 5000 to 7000. The BMI ranged from 28.46 to 29.48 among females across all income quintiles.

### 3.2. Econometric/Statistical Analysis

Table 2 shows the regression results of the BMI–gender relationship along BMI distribution (unconditional distribution). In terms of marital status, results showed that married individuals were likely to have an increasing BMI in the lower and middle quantile points as compared to unmarried individuals. Furthermore, males had a consistently lower BMI across all quantile points. Another important result is that all education levels had a reducing effect on BMI for individuals in the 50th and 75th quantiles. 

Furthermore, there was a persistently higher BMI for employed individuals across all quantiles, which perhaps explains the lack of exercise and diet consciousness among busier people. This was a more surprising result in terms of age, as all age groups across all quantiles showed persistently high BMI. These results are surprising because it was expected that a young age group would be more active, and individuals in this age group would be involved in more physical activities that induce a negative effect on BMI.

The regression analysis for gender–BMI distribution (unconditional distribution) was performed for the 25th percentile (Q25), median (Q50), 75th percentile (Q75), and the 90th percentile (Q90). Individuals belonging to an income quintile between SR 5000 to 7000 and SR 10,000 to 15,000 had a high BMI at the Q25 and Q50 quantile points. Individuals within the SR 7000 to 10,000 income bracket only had a positive increasing effect on BMI at Q25. Results also showed that, in the majority of the regions in the sample, there was a generally lower BMI across all quantiles. In fact, in all regions where the effects were statistically significant, the association was negative. In some regions, there was a negative association between the regions and the outcome of interest across all quantiles, indicating a persistently lower BMI in those regions. In support of Figure 1, the BMI–gender relationship was negative at the 25th, 50th, and 75th percentiles. Having examined the BMI–gender relationship across distribution, we then examined factors explaining the BMI–gender gap. Table 3 indicates aggregate RIF decomposition.

In line with the BMI–gender relationship presented previously, we found that the log BMI for females was higher and significant at *p* < 0.01 than that of males at all quantiles. At Q25, it was 3.168 (*p* < 0.01); at Q50, it was 3.331 (*p* < 0.01); and at Q75, it was 3.48 (*p* < 0.01). The difference in BMI at Q25 (0.9996) was not significant, and it was significant at Q50 (0.0438, *p* < 0.01) and Q75 (0.0627, *p* < 0.01). As can be seen, the difference was higher at Q25, declined at Q50, and increased again at Q75. Much of the difference was explained by the covariate effect at both Q25 (−0.01488, *p* < 0.01) and Q50 (−0.00623, *p* < 0.1), but it was insignificant at Q75. However, we found that the structural effect was significant at all examined quantiles.

From aggregate decomposition, we now examine the detailed decomposition in Table 4 (covariate effect), and the results for the structural effect are available upon request.

Table 4 shows that, in the covariate effect or the explained part, only the variables of employment, the age groups of 25–34 and ≥55 years, and Jazan region consistently explained the difference in distribution at all quantiles. This implies that differences at the lower and upper age quantiles were significant contributors to BMI differences at the various quantiles.

## 4. Discussion

The topic of BMI and its associated factors are perceived as a public health concern only in Western countries. However, the recent rise in income in Asian countries has resulted in lifestyle changes and a rampart rise in non-communicable diseases, which are also loosely referred to as “diseases of the rich” [57,58]. Sedentary and passive lifestyles have become the order of the day, and this has been coupled with the rise of the fast-food industry that has been documented to be one of the factors leading to BMI issues [59]. Using recent SHIS data, the present study uses new distribution-based regression methods to explain the BMI gender gap. The advantage of this method is that we observed heterogeneity on how determinants are associated with BMI differentials at various points of distribution.

The following significant results were obtained. First, there was persistently low BMI in men compared to women across various quantiles of BMI distribution. The existence of gender variation in BMI can be explained by differentials in income at the lower quantiles of BMI distribution, with women gaining increased BMI as income increases as compared to men. Second, the study found that age, education, and employment contributed significantly to the gender difference in the BMI. The RIF decomposition showed that both the covariate effect and the structural effect were significant (*p* < 0.01). Although most of the covariate effect was not significant at the Q75, the structural effect was consistently significant at all the quantiles and increased in magnitude. Third, there was a significant effect of the dietary consumption, such as soda and red meat intake, at the aggregate level, although there were differences for each variable at each quantile. 

Our findings that females have a higher BMI than males across various income quintiles are in close contrast to other studies from the KSA [30] and the USA [60] that found higher BMI in men than in women. However, Al-Qahtani [30] explained that multiple pregnancies can be considered as one of the specific risk factors for increased food intake, coupled with cultural beliefs that women should not practice any form of physical activity until 40 days after delivery, thus leading to weight gain and evidently increasing BMI in women as compared to that in men.

Another interesting finding of the current study is that there is increased BMI among employed people. This result is not surprising because many employed people spend most of their time seated in their offices, and many offices do not have facilities for physical exercise. Moreover, there is excessive use of cars, elevators (as opposed to stairways), and increased calorie intake (and unchecked diets) from foods consumed during office meetings [61]. Indeed, those employed showed a higher BMI, increasing effects for individuals in the lower quantiles [62]. However, some findings from other studies showed a negative relationship between BMI and employment because higher employment is associated with higher education [63]. Moreover, highly educated people have the knowledge and ability to manage their body weight, and they exhibit almost normal weight.

Lastly, a rather surprising result was that, although young people were expected to have lower BMI as this group is deemed to be more active, the obtained results said otherwise. However, this is not a cause for alarm, as results showed that the BMI-increasing effect of age is lower for young males and females than for older people, and this conforms to expected results that young people gain more body mass slower than older people because of their excess activity [64]. This finding also supports the results of previous research that revealed that obesity rates are high among different age groups [65]. Further, an interesting finding was that the positive effect of age on BMI was lower for people in higher quantiles compared to those in lower quantiles; this signifies the importance of knowledge and access to information on body care in people in higher quantiles. 

Finally, some caution should be exercised while interpreting the results of this study. Given that we did not address potential endogeneity or reverse causality that some of the variables might have, it is necessary to view the results as not causal. However, there was nothing we could do in terms of using RIF regression to express the causal mechanism, because existing methods on mean- and distribution-based decomposition techniques do not address such issues. Future studies should consider extending these methods to allow for the use of other techniques, such as instrumental variable extensions, to be incorporated in the analysis.

Although we used BMI as a measure of adiposity, it is also worth mentioning the issues raised against the usage of BMI. First, BMI is limited in its inability to accurately estimate body fat percentage due to misclassification of body fat-defined obesity [8,66]. Second, there are variations in the thresholds; for example, the World Health Organization (WHO) has different measures. Third, the BMI cannot distinguish between lean and fat mass, and provides no indication of body fat distribution [67]. Apart from the highlighted issues regarding the limitations of using BMI as a measure of adiposity, it can also be said that BMI is a less accurate predictor of body fat in the elderly population because they are likely to lose muscle with age [68]. Thus, BMI will not capture muscle loss. Lastly, because the survey did not capture whether the females were pregnant or not at the time of the survey, there is a potential that the BMI may be overstated for those who were pregnant. 

Our study suggests a number of policy implications. The government should consider introducing programs that specifically target women to help them reduce BMI. These programs could include organizing sporting events at the workplace and at the national level. Furthermore, given that we found soda consumption to be another contributing factor, the effect of soda consumption could be reduced by levying a tax on beverages, which might reduce the demand for soda due to the increased price. Given the significance of education in contributing to the difference, it is essential to introduce education programs that teach and encourage people to check their BMI and weight. Lastly, since we found age to be a strong contributor, it may also be essential to perform regular health checks for older people as a good health practice approach.

## 5. Conclusions

This study aimed to find gender differentials in the BMI of people in the KSA. Results showed that there is indeed a difference in BMI between males and females, with females having a persistently higher BMI compared to that of males. Results also showed that various correlations, such as age, employment status, income levels, and education, are significant in explaining the observed BMI differentials. Lower education and incomes levels were associated with a high BMI, which indicates that lack of knowledge on nutrition and the inability to have a balanced and nutritious diet have a larger influence on increased body mass. Additionally, soda and red meat consumption had significant effects on BMI differences. 

## Figures and Tables

**Table 1:** Social and demographic characteristics. Results from the 2013 Saudi Health Interview Survey (SHIS).

Explanatory Variables	Male		Female		Difference	*p*-Value
	Mean	Std. Error	Mean	Std. Error		
Body mass index (BMI)	27.971	11.546	29.123	11.289	1.152	<0.001
Married	0.701	0.458	0.636	0.481	−0.065	<0.001
Education						
Below primary school	0.111	0.314	0.257	0.437	0.147	<0.001
Primary school	0.115	0.319	0.093	0.291	−0.021	0.002
Intermediate school	0.320	0.467	0.246	0.431	−0.074	<0.001
High school	0.195	0.396	0.145	0.352	−0.050	<0.001
College/university +	0.260	0.439	0.259	0.438	−0.001	0.922
Employed	0.625	0.484	0.670	0.470	0.044	<0.001
Age						
15–24	0.197	0.398	0.216	0.411	0.019	0.043
25–34	0.253	0.435	0.284	0.451	0.030	0.003
35–44	0.232	0.422	0.226	0.418	−0.006	0.515
45–54	0.140	0.347	0.141	0.348	0.001	0.933
≥55	0.177	0.382	0.134	0.340	−0.044	<0.001
Monthly income						
<SR 3000	0.142	0.349	0.211	0.408	0.069	<0.001
SR 3000 to less than 5000	0.180	0.384	0.193	0.394	0.013	0.146
SR 5000 to less than 7000	0.168	0.374	0.163	0.370	−0.005	0.593
SR 7000 to less than 10,000	0.201	0.401	0.180	0.384	−0.022	0.015
SR 10,000 to less than 15,000	0.175	0.380	0.143	0.350	−0.032	<0.001
≥SR 15,000	0.134	0.341	0.111	0.314	−0.024	0.001
Days of soda consumption	4.237	2.740	3.681	2.606	−0.556	<0.001
Days of red meat consumption	3.420	1.679	2.896	1.514	−0.523	<0.001
Region						
Almadina Almonawra	0.074	0.262	0.052	0.221	−0.023	<0.001
Albaha	0.066	0.249	0.079	0.270	0.013	0.026
Aljouf/Quriat	0.042	0.201	0.041	0.197	−0.002	0.737
Aseer/Bisha	0.088	0.284	0.094	0.292	0.006	0.375
Eastern Region	0.077	0.266	0.064	0.244	−0.013	0.025
Haiel	0.073	0.260	0.063	0.244	−0.009	0.101
Jazan	0.063	0.243	0.093	0.291	0.030	<0.001
Najran	0.074	0.261	0.080	0.272	0.007	0.264
Northern Borders	0.058	0.234	0.056	0.230	−0.002	0.695
Qaseem	0.031	0.175	0.037	0.190	0.006	0.162
Riyadh	0.142	0.349	0.136	0.343	−0.006	0.430
Tabouk	0.050	0.217	0.049	0.217	−0.000	0.970
Western Region	0.162	0.369	0.155	0.362	−0.007	0.399
Observations	4128		3618			

**Table 2:** Regression results for gender–BMI distribution (unconditional distribution). Results from the 2013 Saudi Health Interview Survey (SHIS).

Explanatory Variables	Q25	Q50	Q75	Q90
Married	0.053 ***	0.023 ***	0.001	−0.026 **
	(0.009)	(0.008)	(0.009)	(0.013)
Male	−0.025 ***	−0.051 ***	−0.067 ***	−0.066 ***
	(0.007)	(0.006)	(0.007)	(0.009)
Education				
Primary school	−0.022	−0.021 *	−0.039 ***	0.003
	(0.013)	(0.012)	(0.014)	(0.020)
Intermediate school	0.014	−0.019 *	−0.049 ***	−0.016
	(0.012)	(0.011)	(0.013)	(0.018)
High school	−0.013	−0.020 *	−0.040 ***	0.002
	(0.013)	(0.012)	(0.014)	(0.019)
College/university +	0.006	−0.029 **	−0.067 ***	−0.027
	(0.012)	(0.012)	(0.014)	(0.018)
Employed	0.042 ***	0.023 ***	0.018 **	0.017
	(0.009)	(0.008)	(0.009)	(0.012)
Age				
25–34	0.135 ***	0.093 ***	0.059 ***	0.060 ***
	(0.014)	(0.011)	(0.011)	(0.015)
35–44	0.201 ***	0.160 ***	0.125***	0.117 ***
	(0.015)	(0.012)	(0.013)	(0.018)
45–54	0.225 ***	0.193 ***	0.150 ***	0.135 ***
	(0.015)	(0.013)	(0.015)	(0.020)
≥55	0.240 ***	0.177 ***	0.12 1***	0.120 ***
	(0.015)	(0.013)	(0.015)	(0.020)
Monthly income				
SR 3000 to less than 5000	0.028 **	0.020 **	0.009	0.007
	(0.012)	(0.010)	(0.011)	(0.016)
SR 5000 to less than 7000	0.042 ***	0.030 ***	0.018	0.010
	(0.013)	(0.011)	(0.012)	(0.017)
SR 7000 to less than 10,000	0.044 ***	0.014	0.001	−0.009
	(0.013)	(0.011)	(0.012)	(0.016)
SR 10,000 to less than 15,000	0.048 ***	0.029 **	0.008	−0.003
	(0.013)	(0.011)	(0.013)	(0.018)
≥SR 15,000	0.042 ***	0.026 **	0.014	−0.028
	(0.014)	(0.012)	(0.014)	(0.018)
No. of days of soda consumption	−0.004	0.002	0.012 ***	0.013 **
	(0.005)	(0.004)	(0.005)	(0.006)
No. of days of red meat consumption	0.015**	0.008	0.007	−0.018**
	(0.007)	(0.006)	(0.006)	(0.009)
Region				
Almadina Almonawra	−0.054 ***	−0.037 ***	−0.040 **	−0.027
	(0.016)	(0.014)	(0.016)	(0.020)
Albaha	−0.044 ***	−0.014	−0.025	0.013
	(0.015)	(0.013)	(0.016)	(0.021)
Aljouf/Quriat	−0.018	0.016	0.023	0.070**
	(0.018)	(0.016)	(0.019)	(0.029)
Aseer/Bisha	−0.064 ***	−0.049 ***	−0.040 ***	−0.007
	(0.014)	(0.013)	(0.014)	(0.019)
Eastern Region	−0.037 **	0.001	−0.015	−0.014
	(0.015)	(0.014)	(0.015)	(0.020)
Haiel	0.015	0.019	0.009	−0.023
	(0.014)	(0.014)	(0.016)	(0.020)
Jazan	−0.095 ***	−0.088 ***	−0.080 ***	−0.057 ***
	(0.016)	(0.013)	(0.014)	(0.018)
Najran	−0.027 *	−0.030 **	−0.025	−0.031
	(0.015)	(0.013)	(0.015)	(0.020)
Northern Borders	−0.047 ***	−0.011	0.001	0.002
	(0.017)	(0.015)	(0.017)	(0.022)
Qaseem	−0.003	0.004	0.004	0.004
	(0.019)	(0.018)	(0.021)	(0.028)
Tabouk	0.016	0.007	−0.002	0.034
	(0.016)	(0.016)	(0.018)	(0.025)
Western Region	−0.059 ***	−0.039 ***	−0.032 **	−0.008
	(0.012)	(0.011)	(0.012)	(0.016)
_cons	2.953 ***	3.200 ***	3.422 ***	3.571 ***
	(0.019)	(0.016)	(0.018)	(0.024)
N = 7746				

Standard errors in parentheses; * *p* < 0.10, ** *p* < 0.05, *** *p* < 0.01. Q25: 25th percentile; Q50: median; Q75: 75th percentile; Q90: 90th percentile.

**Table 3:** Recentered influence function decomposition of BMI distributional difference. Results from the 2013 Saudi Health Interview Survey (SHIS).

Overall Decomposition	Q25	Q50	Q75
Females	3.16788 ***	3.33107 ***	3.48049 ***
	(0.00582)	(0.00492)	(0.00498)
Males	3.15827 ***	3.29070 ***	3.41774 ***
	(0.00461)	(0.00368)	(0.00420)
Difference	0.00961	0.04038 ***	0.06274 ***
	(0.00742)	(0.00614)	(0.00652)
Explained (covariate effect)	−0.01488 ***	−0.00632 *	−0.00536
	(0.00377)	(0.00296)	(0.00288)
Unexplained (structural effect)	0.02449 ***	0.04670 ***	0.06811 ***
	(0.00738)	(0.00620)	(0.00671)

* *p* < 0.10, *** *p* < 0.01.

**Table 4:** Detailed decomposition of explained covariate effect across distribution. Results from the 2013 Saudi Health Interview Survey (SHIS).

Explanatory Variables	Q25	Q50	Q75
Married	−0.00298 ***	−0.00149 *	−0.00008
	(0.00080)	(0.00058)	(0.00056)
Education			
Primary school	0.00061	0.00051	0.00050
	(0.00035)	(0.00031)	(0.00034)
Intermediate school	−0.00041	0.00201 *	0.00149
	(0.00092)	(0.00088)	(0.00097)
High school	0.00115	0.00135 *	0.00047
	(0.00068)	(0.00063)	(0.00068)
College/university +	0.00001	0.00004	0.00004
	(0.00008)	(0.00037)	(0.00039)
Employed	0.00187 **	0.00121 **	0.00090 *
	(0.00062)	(0.00046)	(0.00044)
Age			
25–34	0.00438 **	0.00263 **	0.00170 **
	(0.00152)	(0.00093)	(0.00065)
35–44	−0.00133	−0.00096	−0.00077
	(0.00205)	(0.00147)	(0.00119)
45–54	0.00016	0.00012	0.00010
	(0.00189)	(0.00146)	(0.00116)
≥55	−0.01081 ***	−0.00733 ***	−0.00580 ***
	(0.00215)	(0.00150)	(0.00125)
Monthly income			
SR 3000 to less than 5000	0.00034	0.00028	0.00003
	(0.00028)	(0.00023)	(0.00014)
SR 5000 to less than 7000	−0.00019	−0.00011	−0.00001
	(0.00036)	(0.00022)	(0.00006)
SR 7000 to less than 10,000	−0.00094 *	−0.00031	0.00029
	(0.00048)	(0.00026)	(0.00027)
SR 10,000 to less than 15,000	−0.00153 **	−0.00085 *	−0.00014
	(0.00059)	(0.00041)	(0.00039)
≥SR 15,000	−0.00092 *	−0.00042	−0.00027
	(0.00045)	(0.00031)	(0.00033)
No. of days of soda consumption	0.00064	−0.00046	−0.00160 *
	(0.00073)	(0.00062)	(0.00071)
No. of days of red meat consumption	−0.00304 *	−0.00050	−0.00095
	(0.00125)	(0.00102)	(0.00114)
Region			
Almadina Almonawra	0.00130 **	0.00062	0.00101 *
	(0.00048)	(0.00035)	(0.00042)
Albaha	−0.00062	−0.00003	−0.00035
	(0.00034)	(0.00017)	(0.00025)
Aljouf/Quriat	0.00002	−0.00004	−0.00005
	(0.00007)	(0.00012)	(0.00016)
Aseer/Bisha	−0.00040	−0.00023	−0.00020
	(0.00045)	(0.00027)	(0.00024)
Eastern Region	0.00049	−0.00004	0.00025
	(0.00030)	(0.00017)	(0.00022)
Haiel	−0.00015	−0.00020	0.00005
	(0.00016)	(0.00017)	(0.00015)
Jazan	−0.00288 ***	−0.00235 ***	−0.00208 ***
	(0.00076)	(0.00062)	(0.00059)
Najran	−0.00019	−0.00010	−0.00018
	(0.00020)	(0.00013)	(0.00019)
Northern Borders	0.00009	0.00002	0.00001
	(0.00023)	(0.00006)	(0.00004)
Qaseem	−0.00000	0.00009	0.00005
	(0.00011)	(0.00012)	(0.00012)
Tabouk	−0.00000	−0.00000	−0.00000
	(0.00008)	(0.00009)	(0.00006)
Western Region	0.00045	0.00023	0.00024
	(0.00054)	(0.00028)	(0.00030)

Standard errors in parentheses, * *p* < 0.10, ** *p* < 0.05, *** *p* < 0.01.
